# Estimation of preterm labor immediacy by nonlinear methods

**DOI:** 10.1371/journal.pone.0178257

**Published:** 2017-06-01

**Authors:** Iker Malaina, Luis Martinez, Roberto Matorras, Carlos Bringas, Larraitz Aranburu, Luis Fernández-Llebrez, Leire Gonzalez, Itziar Arana, Martín-Blas Pérez, Ildefonso Martínez de la Fuente

**Affiliations:** 1 Department of Mathematics, University of the Basque Country UPV/EHU, Leioa, Spain; 2 Cruces University Hospital, Obstetrics and Gynecology Department, Barakaldo, Spain; 3 Department of medical-surgical specialties, University of the Basque Country UPV/EHU, Leioa, Spain; 4 Department of Cell Biology and Histology, University of the Basque Country UPV/EHU, Leioa, Spain; 5 Department of Applied Mathematics, Statistics and Operation Research, University of the Basque Country UPV/EHU, Leioa, Spain; 6 Department of Nutrition, CEBAS-CSIC Institute, Espinardo University Campus, Murcia, Spain; University of Washington, UNITED STATES

## Abstract

Preterm delivery affects about one tenth of human births and is associated with an increased perinatal morbimortality as well as with remarkable costs. Even if there are a number of predictors and markers of preterm delivery, none of them has a high accuracy. In order to find quantitative indicators of the immediacy of labor, 142 cardiotocographies (CTG) recorded from women consulting because of suspected threatened premature delivery with gestational ages comprehended between 24 and 35 weeks were collected and analyzed. These 142 samples were divided into two groups: the delayed labor group (n = 75), formed by the women who delivered more than seven days after the tocography was performed, and the anticipated labor group (n = 67), which corresponded to the women whose labor took place during the seven days following the recording. As a means of finding significant differences between the two groups, some key informational properties were analyzed by applying nonlinear techniques on the tocography recordings. Both the regularity and the persistence levels of the delayed labor group, which were measured by Approximate Entropy (ApEn) and Generalized Hurst Exponent (GHE) respectively, were found to be significantly different from the anticipated labor group. As delivery approached, the values of ApEn tended to increase while the values of GHE tended to decrease, suggesting that these two methods are sensitive to labor immediacy. On this paper, for the first time, we have been able to estimate childbirth immediacy by applying nonlinear methods on tocographies. We propose the use of the techniques herein described as new quantitative diagnosis tools for premature birth that significantly improve the current protocols for preterm labor prediction worldwide.

## Introduction

According to the World Health Organization, one tenth of all human births are premature, which translates to about 15 million babies being born preterm (before the 37th gestational week is completed) every year. Complications derived from being born prematurely lead to the death of nearly one million children annually, which makes it the leading cause of child mortality under the age of five. Usually, the cause behind preterm birth remains unknown, but several risk factors such as multiple pregnancy, pre-eclampsia, vascular disease, uterine overdistension, infection, previous preterm births, uterine malformations, smoking, low maternal body-mass and stress have been described [1,2].

In order to detect threatened premature delivery, obstetrical emergency units usually perform a systemic and obstetric examination, a blood analysis, a vaginal fibronectin determination, a vaginal ultrasound and an external cardiotocography (CTG). Although some indicators have been associated with premature delivery [3–5], the available protocols to predict this complication are still far from perfect. In fact, in a review of 22 different tests, it was concluded that their quality and accuracy was generally poor [6]. Current screening tests for the prediction of preterm labor can be divided into three general categories: risk factor assessment, cervical measurement, and biochemical markers [7].

First, prediction based on risk factor assessment alone is unreliable [7]. While, for instance, a previous delivery before the 34^th^ week of pregnancy poses a risk factor of 13 [8], basing the prognosis in such factors alone would lead to misprediction in more than 50% of the cases [7].

Cervical measurement by transvaginal ultrasound is the most employed technique in the prediction of preterm delivery. Specifically, a short cervical length is associated to a relative risk of 6 [3]. A meta-analysis of 28 studies reported sensitivities ranging from 53% to 67% and specificities ranging from 89% to 92% for delivery within one week [9]. However, due to limitations in ultrasound availability and operator expertise, cervical length cannot be universally and reliably utilized to routinely predict preterm labor on its own [7,10].

Lastly, cervicovaginal fetal fibronectin and phosphorylated insulin-like growth factor binding protein-1 (phIGFBP1) are among the most widely utilized biomarkers of preterm delivery, in spite of their shortcomings. A systematic review of 13 studies showed that when fibronectin was used to predict premature labor, its sensitivity and specificity were highly variable, ranging between 23–92%, and 59–97% respectively [11,12]. On the other hand, even if the protein phIGFBP1A is a good negative predictor of preterm birth with a specificity of 90.5–91.8%, lacks of suitable sensitivity (22.2–69.2%) and positive predictive value (11.8–50%) for precise forecasting [13].

In addition to these three important prognosis tools, a crucial indicator of labor beginning and progression is the presence of uterine contractions. In clinical practice, they are evaluated through the recording of the intrauterine pressure by tocography devices. These contractions are characterized by a triple descending gradient: 1) propagation of the contractile wave in a descending direction; 2) longer duration of the contraction at the uterine fundus than in the inferior parts of the organ; 3) stronger contractions in the upper parts of the uterus than in the lower areas [14]. In addition to these physical changes, several biochemical and electrophysiological processes are altered during uterine contractions [15–17]. Nevertheless, even if there is universal agreement concerning the importance of some measures of uterine activity such as frequency, intensity and amplitude of contractions [18], recent studies have indicated that neither studying the frequency [19], nor analyzing conventional quantitative measures [20] (e.g., Montevideo Units [21]) are accurate in order to predict preterm delivery.

Given the limitations of traditional methods, the alternative of using nonlinear techniques was proposed to approach preterm birth analysis detection [22]. For instance, Approximate Entropy (ApEn) [23] has been frequently utilized to analyze biological time series [24]. This method has been used on CTGs to search for differences on the regularity levels of fetal heart rate between pathological and healthy groups [25,26], as well as on electrohysterograms [27], to find differences between labor and pregnancy contractions.

On the other hand, the calculation of the Hurst coefficient [28], which is most commonly used to detect long-term memory in time series, has also been able to identify anomalies in fetal heart rates intrapartum [29] and to discriminate between healthy and Intrauterine Grow Retarded Fetuses [30]. Later, Hurst proposed a generalization of his method, making it capable of analyzing the persistence level at different scales.

Since labor occurs as a consequence of the combination of increased longer-lasting depolarizations, raised myocyte to myocyte connectivity, and the activation of intracellular contractile machinery [31], we hypothesized that some subtle changes in uterine dynamics (small modifications in the myocyte to myocyte coordination pattern) as well as in cell to cell electrophysiological alterations (specially changes related to bursting-type action potentials) [17] could have an impact on preterm delivery, and that they could be ascertained in conventional cardiotocograms through a variability analysis of uterine pressure recordings.

Here, we have used nonlinear techniques to estimate the immediacy of labor on women with suspected threat of premature delivery through the analysis of the CTGs, which are universally utilized in medical practice [18]. Approximate Entropy and Generalized Hurst Exponent have been applied to the series obtained by taking the increments in the tocographies, and we have found that both statistics were significantly different between women whose labor occurred more than seven days after the tocography recording and women who gave birth during those seven days.

In this paper, for the first time, we have found significant differences on the persistence and regularity levels of the tocographies of women suspected of threatened premature delivery depending on the remaining time until childbirth, which offers the possibility of a new quantitative diagnosis tool.

## Materials and methods

### Sample acquisition and processing

During a four-year period (2010–2013), 1,643 women were assisted at Cruces University Hospital (Basque Country) because of suspected threat of premature delivery (STPD). This state was determined when a pregnant woman was admitted to the obstetrical emergency unit with a gestational age comprehended between 24.0 and 37.0 weeks (based on the last menstrual period and vaginal ultrasound), because of any of the following causes: a) self-reported regular uterine contractions, b) intermittent abdominal pain after excluding other pathological conditions, or c) self-reported expulsion of amniotic fluid.

Two conditions were required to confirm the threatened premature delivery. First, the recording of at least 4 uterine contractions, each lasting at least 30 seconds long at the tocography in a 30 minute period, or 8 uterine contractions in a 60 minute period. Second, a cervical effacement≥ 80% and at the same time, a cervical dilatation≥ 2 cm; in cases with cervical effacement < 80% and/or cervical dilatation < 2 cm, a cervical shortening < 25 mm at 24–31 weeks, or < 15 mm at 32–34 weeks was required. In cases where threatened premature delivery was confirmed and the gestational age was comprehended between 24.0 and 35.0 gestational weeks (or 34.0 if membranes are ruptured), treatment was applied, with the oxytocin receptor blocker atosiban (Tractocile, Ferring Pharmaceuticals A/S, Copenhagen, Denmark).

To avoid the effect of any previous medication, we restricted the study to the first 30 minutes of the tocographies (thus obtaining a single recording of approximately 30 minutes for each patient, which were measured indistinctively by Philips Avalon FM30, Philips Avalon FM20, Hewlett Packard Vidria 50XM and Hewlett Packard 50IP cardiotocographs) performed on the initial consultation for STPD of the women whose gestational age was less than 35.0 weeks (or 34.0 if membranes were ruptured), because over this gestational age no treatment is applied, on the basis of the excellent newborn outcome. Then, two specific subgroups were considered: a) the delayed labor group, constituted by those women whose labor took place more than seven days after the initial consultation and b) the anticipated labor group, constituted by all the women whose delivery occurred in the following seven days. During the analysis period, 75 cases belonging to the delayed labor group were documented. Afterwards, a simple random sampling procedure (i.e., a blind selection from a bigger set) was performed to select 78 cases for the anticipated labor group. After being analyzed by a gynecologist, 11 of them were discarded because labor was induced, leaving 67 (24 of which were cesarean sections) cases for the anticipated labor group.

The participants provided their written consent, and both this study and the consent procedure were approved by our center investigation board (Comité Ético de Investigación Clínica del Hospital Universitario Cruces, CEIC-E16/13).

Then, the full signals obtained from the toco transducer were digitized by Engauge Digitizer 4.0 software, an open source program. To maintain the original proportions, the Cartesian coordinate system origin was placed on the first square, that is, the one on the south-west of the measurement, and the length of a square of the tocography was considered to be the unit. Next, the data were discretized to obtain approximately 2,000 time points, equidistant by 0.0291457 units. Besides, the sampling frequency (0.904Hz) was maintained in all the cases, even in those which were slightly shorter than 30 minutes. The time series of Delayed Labor group and Anticipated Labor group are uploaded in S1 and S2 Files, respectively.

In addition to the tocography recording, mothers’ age, gestational age, number of fetuses and mean of the fetuses’ weights (meaning that in case of multiple labors, the individual weights, which were estimated by ultrasounds, were averaged) were collected.

### Approximate Entropy

Approximate Entropy (ApEn) is a measure first proposed by Pincus in 1991 [23], which quantifies the regularity and predictability of a time series. A regular signal containing repetitive patterns has a low ApEn, while a complex and hardly predictable series has a high ApEn. The statistic is calculated as follows:

1. Take a time series of *N* equally spaced values: *U* = *u*(1), *u*(2), …, *u*(*N*). The size of the time series can be relatively small (*N*>100) [32].
2. Fix a positive number *r*, and an integer number *m*. The number *r* specifies a filtering level, and a common choice for this parameter is *r* = 0.2·*SD*, where *SD* is the standard deviation of the signal. The optimal value of *m*, the statistic that represents the length of compared runs of data, was estimated by the method proposed by L.Cao [33], and in our case it resulted in *m* = 2.
3. Form a sequence of vectors *x*(1), *x*(2), …, *x*(*N* − *m* + 1) in ℝ^*m*^, real m-dimensional space, defined by *x*(*i*) = [*u*(*i*), …, *u*(*i* + *m* − 1)].
4. For each *i* = 1, …, *N* − *m* + 1, the sequence *x*(1), *x*(2), …, *x*(*N* − *m* + 1) is used to construct
  $$C_{i}^{m}\left(r\right)=\frac{number\;of\;x\left(j\right)such\;that\;d\left[x\left(i\right),\;x\left(j\right)\right]\leq r}{\left(N-m+1\right)},$$ (1)
  where *d*[*x*(*i*),*x*(*j*)] is defined as
  $$\underset{0\leq k\leq m-1}{\text{max}}\left\{\left|u\left(i+k\right)-u\left(j+k\right)\right|\right\}.$$ (2)
  Thus, *d* represents the distance between the scalar exponents of the vectors *x*(*i*) and *x*(*j*).
5. Then, the variable
  $$\Phi^{m}\left(r\right)={\left(N-m+1\right)}^{-1}\sum_{i=1}^{N-m+1}\text{ln}\left(C_{i}^{m}\left(r\right)\right)$$ (3)
  is established, where ln is the natural logarithm.
6. Finally, Approximate Entropy is defined by
  $$ApEn\left(U,r,m\right)=\Phi^{m}\left(r\right)-\Phi^{m-1}\left(r\right)$$ (4)
  for fixed *m* and *r*.

### Generalized Hurst Exponent

The calculation of the Hurst exponent is a classical method to detect long-term correlations in time series, introduced by the hydrologist H.E. Hurst in 1951 to study the annual discharges of the Nile River [28]. The Hurst exponent *H*, is referred to as the index of long-range dependence, which characterizes how the variance depends on a time interval. Later, Hurst proposed its generalization, the Generalized Hurst Exponent (GHE), which enabled to study the regularity at different levels.

To calculate this statistic, Barabási and Vicsek studied the scaling properties of the data via the *q*th-order moments of the distribution of the increments [34]. Recently Di Matteo revised this method [35], summarized as follows:

1. Take a time series of *N* equally spaced values: *U* = *u*(1), *u*(2), …, *u*(*N*).
2. Define
  $$K_{q}\left(\tau \right)=\sum_{t=0}^{N-\tau }\frac{{\left|u\left(t+\tau \right)-u\left(t\right)\right|}^{q}}{\left(N-\tau +1\right)}$$ (5)
  where *τ* defines the boundary of the analyzed time interval.
3. This statistic scales as *K*_*q*_ (*τ*)~*cτ*^*qH*(*q*)^, where *H(q)* is the *q*th-order Generalized Hurst Exponent (GHE(*q*)).

In this paper, we focus on *q* = 1 (GHE(1)), which describes the scaling behavior of the absolute values of the increments, and on *q* = 2 (GHE(2)), which is related to the scaling of the autocorrelation function and the power spectrum [36].

### Power spectral analysis

Spectral Analysis is used to study the properties of a time series in the frequency domain. It can also determine if the series are described by a fractional Gaussian noise (fGn) or a fractional Brownian motion (fBm) by calculating the slope of the Power Spectral Density (PSD) plot [37]. The signal is said to exhibit power law scaling if the relationship between its Fourier spectrum and the frequency is approximated asymptotically by *S*(*f*) ≈ *S*(*f*_0_)/*f*^*β*^, where *S(f*_*0*_*)* and *β* are constant values. If -1<*β*<1 the signal corresponds to a fGn. When *β = 0* the power spectrum is flat and the time series is composed of a sequence of independent random values, which is the case of white noise. If 1<*β*<3 the signal corresponds to a fBm. These two behaviours can be related by the calculation of the increments (that is, the first differences) of a fractional Brownian motion, which transforms the time series into a fractional Gaussian noise.

### Predictive parameters

The forecasting capacity of an indicator was evaluated by the classical measures of the diagnostic tests:

$$sensitivity=\frac{number\;of\;true\;positives}{number\;of\;true\;positives+number\;of\;false\;negatives}\;,$$ (6)

$$specificity=\frac{number\;of\;true\;negatives}{number\;of\;true\;negatives+number\;of\;false\;positives}\;,$$ (7)

$$positive\;predictive\;value=\frac{number\;of\;true\;positives}{number\;of\;true\;positives+number\;of\;false\;positives}\;,$$ (8)

$$negative\;predictive\;value=\frac{number\;of\;true\;negatives}{number\;of\;true\;negatives+number\;of\;false\;negatives}\;,$$ (9)

## Results

**Fig 1:**
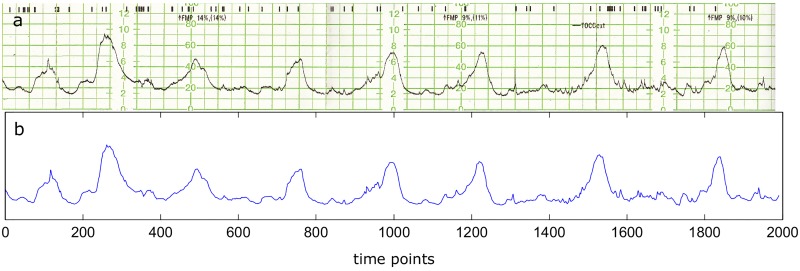
Digitization process applied to a generic tocography. (a) Scanned generic tocography recording (delayed labor group, tocography n°1) of 30 minutes of duration. (b) Digitization of the same tocography by Engauge Digitizer 4.0, represented by 1991 time points.

The objective of this study is to evaluate if there are any informational differences between the tocography recordings depending on the immediacy of the labor. For this purpose, we have analyzed 142 cases of suspected threatened premature delivery with gestational ages comprehended between 24.0 and 35.0 weeks, subdividing them into two groups: the ones who gave birth after at least seven days since their visit (delayed labor, n = 75) and the ones who gave birth in seven days or less since their visit (anticipated labor, n = 67). Then, their tocographies were digitized, allowing us to obtain time series of approximately 2,000 time points. In Fig 1, a representative tocography and the respective digitization are illustrated.

First, in order to find whether significant differences exist between the quantitative variables of the two groups, mothers’ age, gestational age, number of fetuses and mean of the fetuses’ weights were compared. To check whether these quantitative variables were normally distributed or not, we applied the Kolmogorov-Smirnov normality test, which rejected the normality hypothesis in all cases. Thus, a non-parametric test, the Wilcoxon rank-sum test, was performed to compare the variables of both populations. In all the cases, the null hypothesis could not be rejected because the p-values were greater than 0.05, indicating that these variables were unable to distinguish between the distributions of the delayed and the anticipated labor group. The medians±iqr (interquartile range, Q3-Q1) and the p-values of the respective Wilcoxon rank-sum test for both groups are given in Table 1. In addition, to test if one group presented more uterine activity than the other, we calculated and compared the standard deviation of the time series obtained from the CTGs. The median value for the delayed labor group was 0.755 with a interquartile range of 0.72, and for the anticipated labor group was 0.725 (iqr = 0.68), with a p-value of 0.381 on the rank-sum test, which indicates that there were no significant differences between both groups.

**Table 1:** Classical obstetric variables.

	mothers’ age (years)	gestational age(days)	n° fetuses	mean of the fetuses’ weights (g)
**Delayed Labor**	34±5.75	214±35.5	1±0	1588±837
**Anticipated Labor**	34±5.75	219±30	1±0.75	1740±830
**p-value of W. r. test**	0.991	0.42	0.781	0.801

The data correspond to the medians±iqr (interquartile range) of both groups for mothers’ age (in years), gestational age (in days), number of fetuses and mean of the fetuses’ weights (i.e., the average in grams of the fetuses’ weight of each child, estimated by ultrasounds). The last row corresponds to the p-values of the Wilcoxon rank-sum test performed to discriminate between both groups.

**Fig 2:**
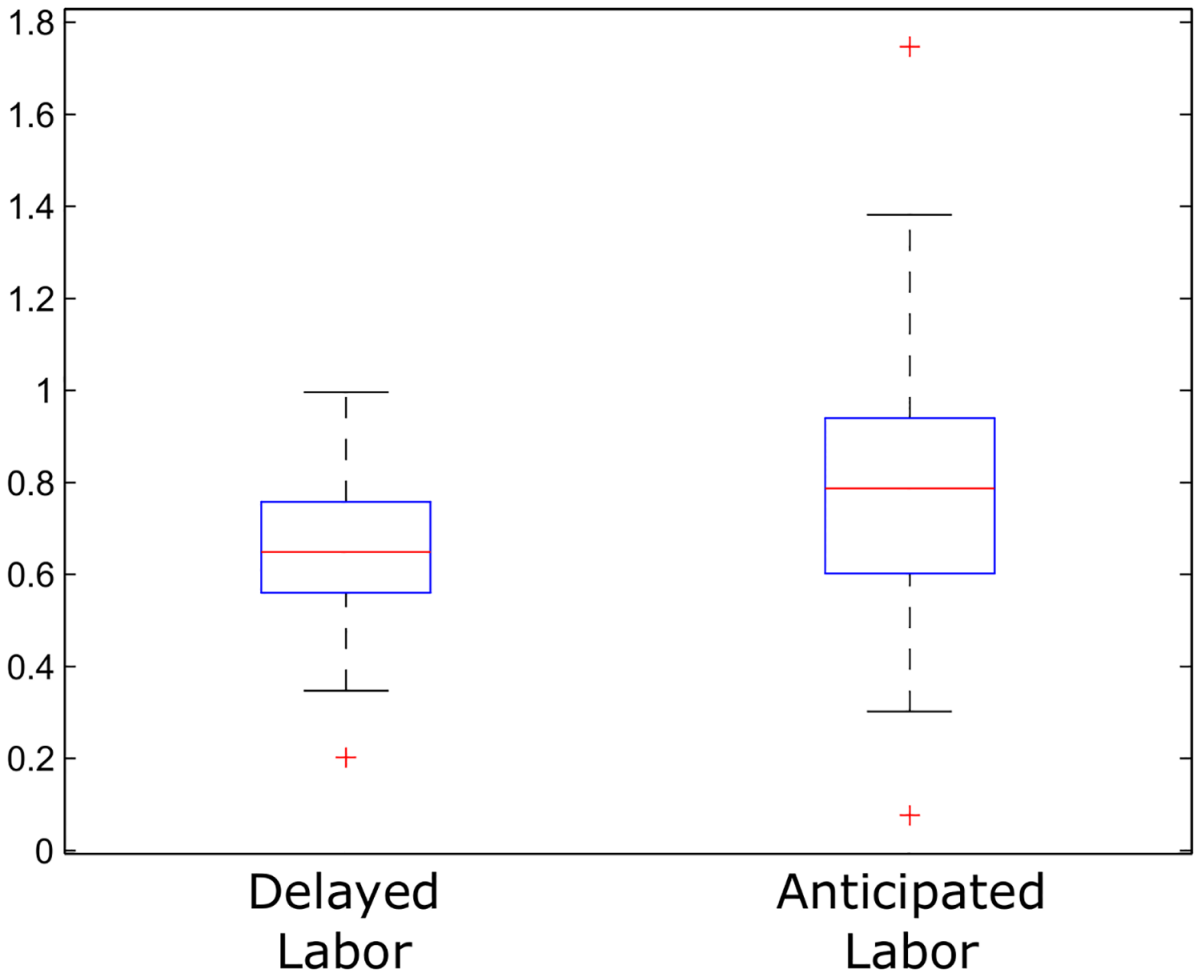
Box plot of the ApEn applied to delayed and anticipated labor groups. Box plot illustration of the distributions of the Approximate Entropy values calculated for the increments of the delayed and the anticipated labor group. The blue boxes represent the distribution of the central 50% of the values and the red lines represent the medians. The rest of the values are represented by the arms, or in the case of atypical values, by red crosses. As can be observed in the figure, the distribution of the values for the delayed and the anticipated labor groups were significantly different.

Next, to quantify the unpredictability of the two subgroups, the Approximate Entropy (ApEn) of every time series was estimated (this entropy was chosen over other entropy measures such as Sample entropy [38] and Conditional entropy [39] because it gave the best results in preliminary studies). To obtain this statistic, the time series were detrended, by calculating the increments (i.e. *u*′(*i*) = *u*(*i* + 1) − *u*(*i*), *i* = 1, …, *N* − 1) and ApEn(*U'*,*m*,*r*) was computed for *m* = 2 and *r* = 0.2·*SD* (for more details see subsection 2 of Material and methods). By studying the nonlinear measures in the increments instead of in the raw signal, we also avoided the necessity to calibrate the baselines and amplitudes of the tocographies. The median value for the delayed labor group was 0.649 (iqr = 0.2), while for the anticipated labor group was 0.787 (iqr = 0.34). Then, we tested if significant differences existed between the delayed labor and the anticipated labor group, by applying the Wilcoxon rank-sum test (because the normality hypothesis for ApEn values was rejected) which gave a p-value of 0.0049, indicating that the two populations are samples from continuous distributions with different medians, at the 1% significance level. These results suggest that the increments of the delayed labor group are less complex and more predictable than the ones of the anticipated labor group. In Fig 2, we illustrate this difference by a box plot, where the quartiles of each group are represented.

**Fig 3:**
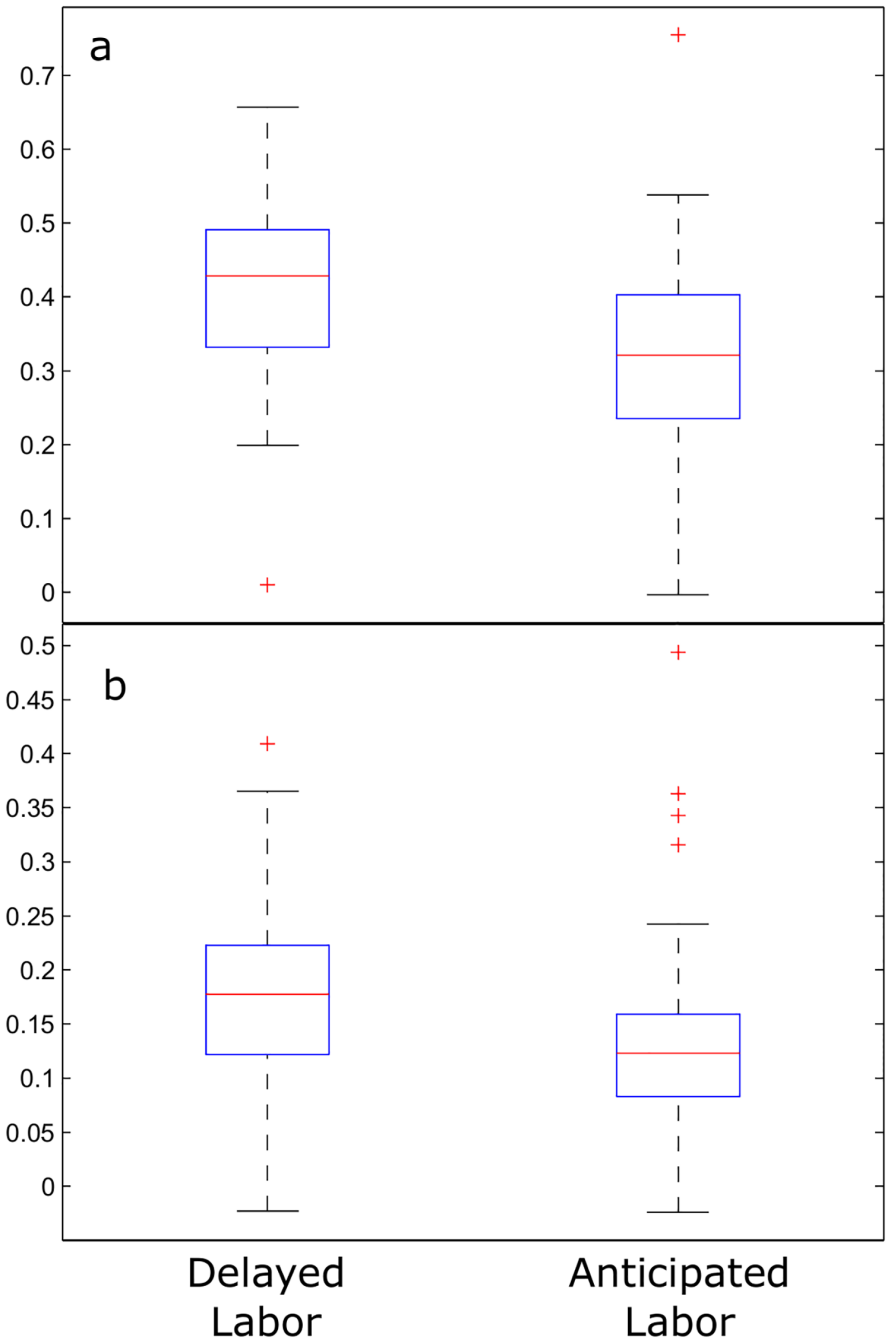
Box plot of the GHE of first and second order applied to delayed and anticipated labor groups. (a) Box plot representation of the distributions of the first order Generalized Hurst Exponent values calculated for the increments of the delayed and the anticipated labor groups. (b) Box plot illustration of the second order GHE distributions of values calculated for the increments of the delayed and anticipated labor groups. In both cases, significant differences between both groups can be observed.

Moreover, we examined whether the groups could be distinguished by the Generalized Hurst Exponent (GHE), and in consequence, by the irregularity. The analysis was performed for the first and second order moments, on the increments of the time series. The median value of the GHE(1) for the delayed labor group was 0.428 (iqr = 0.16), and for the anticipated labor group was 0.321 (iqr = 0.17). In the case of GHE(2), the median value for the delayed labor group was 0.178 (iqr = 0.1) while the mean value for the anticipated labor group was 0.123 (iqr = 0.08). The distributions were compared by a Wilcoxon rank-sum test, and the p-values were 0.000001 and 0.00007 respectively, indicating that the two groups show significantly different regularity levels. To illustrate these differences, in Fig 3, the box plot of the GHE(1) and GHE(2) for the delayed labor and anticipated labor groups are represented.

Then, a spectral analysis was performed to study if both groups could be distinguished by the analysis of their frequency domain. We calculated the Power Spectral Density (PSD) and the slope *β* of the increments, and found that the median value of *β* for the delayed labor group was -0.816 (iqr = 0.52) while the median value for the anticipated labor group was -0.621 (iqr = 0.41). The distributions were significantly different (p-value of 0.00005 on a Wilcoxon rank-sum test). The mean values, the standard deviation and the p-values of the respective Wilcoxon rank-sum test for both groups of ApEn, GHE(1), GHE(2) and PSD slope are shown in Table 2.

**Table 2:** Informational measures.

	ApEn	GH1	GH2	PSD
**Delayed Labor**	0.649±0.2	0.428±0.16	0.178±0.1	-0.816±0.52
**Anticipated Labor**	0.787±0.34	0.321±0.17	0.123±0.08	-0.621±0.41
**p-value of W. r.test**	0.0049	0.000001	0.00007	0.00005

The data correspond to the median±interquartile range of both groups for Approximate Entropy, Generalized Hurst Exponent of first order, Generalized Hurst Exponent of second order and Power Spectral Density slope. The last row corresponds to the p-values of the Wilcoxon rank-sum test performed to discriminate between both groups.

As a means of verifying whether the Approximate Entropy and the Generalized Hurst Exponent values were related to the mothers’ age, the gestational age, the number of fetuses, the mean of the fetuses’ weights, the standard deviation or the PSD slope, a correlation test based on Pearson correlation coefficient was performed. A significant correlation was found (p-value of 7.8 e-37 on the correlation test) between GHE(2) and the PSD slope, with a -0.8270 correlation coefficient. This relationship was expected (for more details see subsection 3 of Material and methods) and has been documented on previous works [36]. Apart from that, all the correlation hypotheses between the classical variables and ApEn or GHE were rejected, except the one correlating the GHE(2) with the mean of the fetuses’ weights, which had a p-value of 0.027 and a correlation coefficient of -0.209. However, this observation was not deepened because several values of the fetuses’ weights were not reported, and the p-value obtained was close to the 0.05 cutoff, questioning its significance.

**Fig 4:**
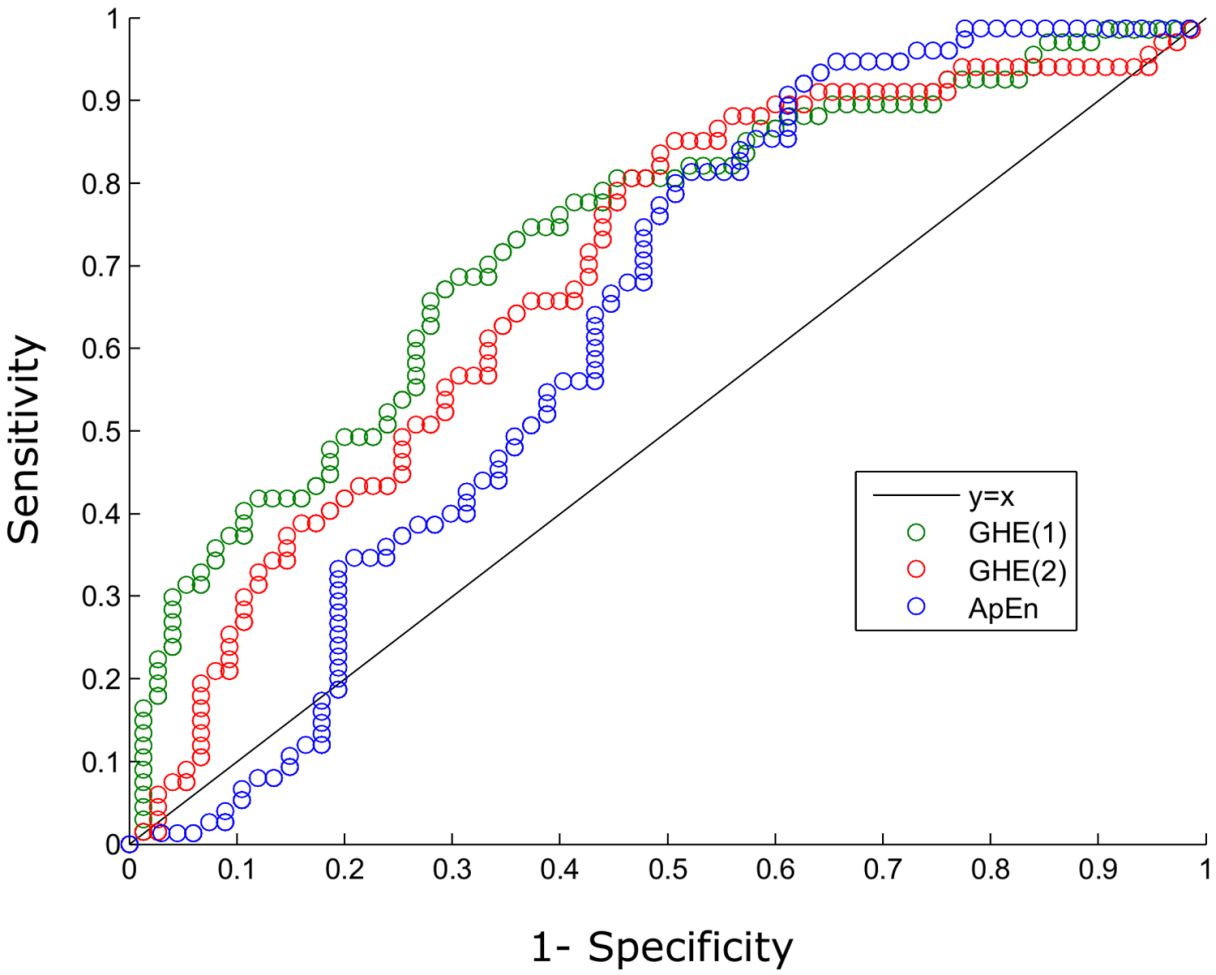
ROC curve of the GHE of first and second orders and the ApEn. The Y axis represents the sensitivity, and the X axis 1-specificity. The green points represent the values for GHE(1) for different thresholds, the red points depict the values for GHE(2) and the blue points represent the values for ApEn. Dots above the oblique black line indicate good balance between sensitivity and specificity. Here, we can observe that no indicator outperforms the rest through the entire curve.

Next, we calculated the sensitivities, specificities, positive predictive values (PPV) and negative predictive values (NPV) of GHE(1), GHE(2) and ApEn used as individual predictors. To calculate these statistical measures, we had to fix a threshold to discriminate between both groups, and in this paper, in order to get a good balance between sensitivity and specificity, we have chosen the threshold that maximizes the mean between both measures, which in the case of GHE(1) was 0.361. Considering the value of the indicator as a test to confirm preterm labor in less than seven days, a positive outcome was associated to GHE(1) values below the threshold, and negative outcome was associated to values above 0.361. Thus, we determined that the sensitivity of the test was 0.687, while the specificity was 0.707. The positive predictive value was 0.676, while the negative predictive value was 0.716. Analogously, the threshold for GHE(2) was set to 0.175 (where lower values were also associated to positive outcome, and higher values to negative outcome), leading to a sensitivity of 0.851, a specificity of 0.507, a PPV of 0.606, and a NPV of 0.792. Then, we calculated the threshold for the ApEn, which was set to 0.891. Higher values were associated to labor before seven days, whereas lower values indicated labor after those seven days. This indicator had a sensitivity of 0.388, a specificity of 0.92, a PPV of 0.813, and a NPV of 0.627. To illustrate the effect of varying the threshold on the sensitivity and the specificity, in Fig 4, the receiver operating characteristic (ROC) curves [40,41] of GHE(1), GHE(2) and ApEn are represented, which had an Area Under the Curve (AUC) of 0.72, 0.678 and 0.634, respectively.

Furthermore, we calculated the performance of the combination of our indicators by evaluating the predictive capacity of two individual predictors simultaneously. We performed three combinations (i.e., GHE(1)&ApEn, GHE(2)&ApEn and GHE(1)&GHE(2)) and calculated the sensitivity, specificity, positive predictive value and negative predictive value in the subsets where the two statistics were simultaneously affirmative or simultaneously negative (which lead to a reduction in the number of evaluated cases). Since two thresholds are needed to evaluate these combinations, we used the ones fixed previously for individual predictors, which were 0.361 for GHE(1), 0.175 for GHE(2) and 0.891 for ApEn. The number of evaluated cases, the positive outcome criterion and the four predictive parameters for GHE(1), GHE(2), ApEn, GHE(1)&ApEn, GHE(2)&ApEn and GHE(1)&GHE(2) are shown in Table 3.

**Table 3:** Informational predictive parameters.

	positive outcome	sensit.	specif.	PPV	NPV
**GHE(1) (n = 142)**	≤0.361	0.687	0.707	0.676	0.716
**GHE(2) (n = 142)**	≤0.175	0.851	0.507	0.606	0.792
**ApEn (n = 142)**	≥0.891	0.388	0.920	0.813	0.627
**GHE(1)&ApEn (n = 96)**	≤0.361 // ≥0.891	0.561	0.927	0.852	0.739
**GHE(2)&ApEn (n = 77)**	≤0.175 // ≥0.891	0.735	0.860	0.806	0.804
**GHE(1)&GHE(2) (n = 114)**	≤0.361 // ≤0.175	0.833	0.633	0.672	0.809

On the first column, the predictive indicator, being GHE(1) and GHE(2) the Generalized Hurst Exponents of first and second order respectively, ApEn the Approximate Entropy, GHE(1)&ApEn the combination evaluating simultaneously the Generalized Hurst Exponent of first order and the Approximate Entropy, GHE(2)&ApEn the combination evaluating simultaneously GHE(2) and ApEn, and GHE(1)&GHE(2) the combination evaluating simultaneously the Generalized Hurst Exponent of fist and second order, with the corresponding number of evaluated cases into brackets. The second column depicts the values associated with a positive outcome, and the rest of the columns represent the sensitivity, specificity, positive predictive value (PPV) and negative predictive value (NPV) for each indicator, respectively.

Then, our results were compared to some classical predictive parameters currently used by obstetricians and gynecologists to predict preterm delivery, such as the vaginal fibronectin, the cervical length and the Bishop score. For this purpose, since in our analysis no classification by gestational age was made (due the shortage of cases), we compared our results to the average of the predictive values for different gestational periods (comprehending from 22 to 35 gestational weeks) obtained from [42] (nonetheless, we would like to remark that our forecast of preterm birth is considered within a range of seven days, while the values of [42] correspond to spontaneous birth). These classical indicators showed higher specificity and negative predictive value than our informational indicators, while the sensitivity and the positive predictive values were lower (the mean±*SD* of these predictive values are given in Table 4). Hence, to contrast the forecasting capacity globally, we calculated the average of the sensitivity and the specificity for each indicator, which gave: 0.606 for vaginal fibronectin, 0.716 for cervical length, 0.658 for Bishop score, 0.697 for GHE(1), 0.679 for GHE(2), 0.654 for ApEn, 0.744 for GHE(1)&ApEn, 0.798 for GHE(2)&ApEn and 0.733 the GHE(1)&GHE(2).

**Table 4:** Classical predictive parameters [4,2].

	positive out.	sensitivity	specificity	PPV	NPV
**v. fibronectin**	≥ 50 ng/mL	0.272±0.12	0.94±0.01	0.318±0.03	0.921±0.03
**c. lenght**	≤ 25 mm	0.611±0.19	0.821±0.07	0.276±0.08	0.949±0.03
**Bishop score**	≥ 4	0.546±0.25	0.769±0.15	0.229±0.11	0.937±0.04

On the first column, the predictive indicators: vaginal fibronectin, cervical length and Bishop score. The second column corresponds to the values associated with a positive outcome, and the rest of the columns represent the mean ± standard deviation of sensitivity, specificity, positive predictive value (PPV) and negative predictive value (NPV) for each indicator respectively, obtained by averaging the values from [42].

Finally, in order to evaluate the predictive capacity of our indicators, we performed a cross-validation analysis by randomly dividing the cases into training data (75% of the CTGs of each group) and test data (the remaining 25% of the CTGs of each group). Then the optimal thresholds were calculated on the training group, and the sensitivity, specificity, positive predictive value, and negative predictive value were estimated on the test group. This operation was repeated 10,000 times, and we obtained, for the GHE(1), an optimal threshold of 0.364±0.02, a sensitivity of 0.657±0.13 (mean±SD), a specificity of 0.668±0.12, a PPV of 0.659±0.08 and a NPV of 0.679±0.07; for the GHE(2), an optimal threshold of 0.165±0.02, a sensitivity of 0.759±0.15 (mean±SD), a specificity of 0.518±0.12, a PPV of 0.599±0.06 and a NPV of 0.713±0.1; lastly for the ApEn, an optimal threshold of 0.863±0.04, a sensitivity of 0.386±0.11, a specificity of 0.86±0.1, a PPV of 0.741±0.12 and a NPV of 0.598±0.04.

## Discussion

Here, in order to find quantitative differences related to the immediacy of labor, we have studied the tocographies of women who attended the hospital because of suspected threatened preterm delivery. The recordings were divided into two groups, depending on the remaining time until labor (more than seven days, and seven days or less since the date the CTG was recorded), and have been analyzed by nonlinear techniques.

First, we compared both groups by classic clinical variables such as mothers’ age, gestational age, number of fetuses, or mean of the fetuses’ weights. In order to evaluate an approximation of the uterine activity, the comparison between the standard deviation of the time series was also made. No significant differences were found, implying that the groups were indistinguishable by those variables.

Second, to evaluate if both groups could be differenced by their predictability, Approximate Entropy was estimated on the increments of every time series. Results have highlighted significant differences (p-value 0.0049) between the anticipated and the delayed labor group. The ApEn values calculated on the anticipated labor group were higher than the ones of the delayed labor group, implying that the increments of the tocographies became more unpredictable in cases where labor finally took place anticipatedly. This indicates that the variations of the tocographies are more irregular, which suggests that cases of anticipated delivery could have a more irregular uterine activity pattern.

Third, we calculated the Generalized Hurst Exponents of first and second order. Significant differences between groups have been found for both measures (with a p-value of 0.000001 and 0.00007, respectively). The low p-values indicate that the two groups behaved very differently according to their regularity, and suggest that this statistic is sensitive to the immediacy of labor. Moreover, first and second order GHE values decreased as labor approached, which could be an indicative of labor proximity.

Then, to confirm our results, the relationship between GHE(2) and Power Spectral Density slope was analyzed. As expected, the low p-value (7.8 e-37) on the correlation test corroborated this dependence. Besides, we demonstrated that neither ApEn nor GHE were correlated to the classical quantitative variables documented in obstetric units.

Next, we evaluated the predictive capacity of the Generalized Hurst Exponent of first and second orders, and of the Approximate Entropy individually, by calculating the sensitivity, specificity, positive and negative predictive values. In the case of GHE, all the diagnostic parameters were near 0.7, indicating that by just considering 30 minutes of tocography, this statistic alone discriminated correctly 70% of the cases in both groups. Additionally, we studied the predictive properties of our indicators evaluated simultaneously in pairs. Between the three combinations, the one considering the Generalized Hurst Exponent of second order and the Approximate Entropy achieved the highest average of sensitivity and specificity. By evaluating this two indicators at once, almost all the diagnostic parameters increased considerably (compared with GHE(1) alone), being close to 0.8. However, even if this simultaneous evaluation achieved better predictive parameters than individual indicators, it had the disadvantage of only being applicable when both statistics agreed on the outcome. As a consequence, the number of cases in which GHE(2)&ApEn could be used was smaller than the number of cases in which individual measures could be applied. Even so, since the objective of this work is to obtain the best predictors in order to improve preterm delivery prediction, we suggest the use of GHE(2)&ApEn when the diagnosis of the GHE(2) and the ApEn coincides, and the use of GHE(1) when their forecasts differ.

We acknowledge that even if the performance in the cross-validation analysis was similar to the one within sample, and some of the predictive parameters of our indicators were remarkable compared to other techniques present in the literature, these results need to be confirmed in prospective trials, one of which is currently being carried out by our group. Moreover, we are gathering data from women suspected of threatened premature delivery with gestational ages comprehended between 35.0 and 37.0 weeks, to test if these predictive techniques are also capable of discerning between groups during this labor period.

In brief, we have analyzed some of the key informational properties of external tocographies. As a means of estimating the immediacy of labor on preterm delivery cases, we have searched for significant differences between women whose labor occurred more than seven days after the tocography recording, and women who gave birth during those seven days, by applying nonlinear methods. In this work, we have found that both Approximate Entropy and Generalized Hurst Exponent values were significantly different between the anticipated and the delayed labor group. Due to the high statistical significance of our results and the predictive capacity of our indicators, we propose the use of these techniques as new quantitative diagnosis tools for premature birth, that significantly improve the current methodology of preterm labor detection worldwide.

## Supporting information

S1 File Delayed labor time series. This file contains the time series of the Delayed Labor group in.m format (Matlab).
(MAT)

S2 File Anticipated labor time series. This file contains the time series of the Anticipated Labor group in.m format (Matlab).
(MAT)
